# Classical Estrogen Receptors and ERα Splice Variants in the Mouse

**DOI:** 10.1371/journal.pone.0070926

**Published:** 2013-08-05

**Authors:** Debra L. Irsik, Pamela K. Carmines, Pascale H. Lane

**Affiliations:** 1 Department of Cellular and Integrative Physiology, University of Nebraska Medical Center, Omaha, Nebraska, United States of America; 2 Department of Pediatrics, University of Oklahoma Health Sciences Center, Oklahoma City, Oklahoma, United States of America; University of Navarra School of Medicine and Center for Applied Medical Research (CIMA), Spain

## Abstract

Estrogens exert a variety of effects in both reproductive and non-reproductive tissues. With the discovery of ERα splice variants, prior assumptions concerning tissue-specific estrogen signaling need to be re-evaluated. Accordingly, we sought to determine the expression of the classical estrogen receptors and ERα splice variants across reproductive and non-reproductive tissues of male and female mice. Western blotting revealed that the full-length ERα66 was mainly present in female reproductive tissues but was also found in non-reproductive tissues at lower levels. ERα46 was most highly expressed in the heart of both sexes. ERα36 was highly expressed in the kidneys and liver of female mice but not in the kidneys of males. ERβ was most abundant in non-reproductive tissues and in the ovaries. Because the kidney has been reported to be the most estrogenic non-reproductive organ, we sought to elucidate ER renal expression and localization. Immunofluorescence studies revealed ERα66 in the vasculature and the glomerulus. It was also found in the brush border of the proximal tubule and in the cortical collecting duct of female mice. ERα36 was evident in mesangial cells and tubular epithelial cells of both sexes, as well as podocytes of females but not males. ERβ was found primarily in the podocytes in female mice but was also present in the mesangial cells in both sexes. Within the renal cortex, ERα46 and ERα36 were mainly located in the membrane fraction although they were also present in the cytosolic fraction. Given the variability of expression patterns demonstrated herein, identification of the specific estrogen receptors expressed in a tissue is necessary for interpreting estrogenic effects. As this study revealed expression of the ERα splice variants at multiple sites within the kidney, further studies are warranted in order to elucidate the contribution of these receptors to renal estrogen responsiveness.

## Introduction

Although estrogen was discovered in the 1920s [1], [2] and the hormone was proposed to act via a receptor, the human estrogen receptor (ERα66) was not cloned until 1986 [3]. Subsequent advances in the understanding of estrogen signaling came with the development of the estrogen receptor knockout mouse [4], the identification and cloning of a second estrogen receptor, ERβ [5], [6], and development of its knockout mouse [7]. Through these studies, the existence of multiple receptors for estrogen signaling with unique downstream events became evident. Novel roles for estrogens have been demonstrated in non-reproductive tissues, such as the skeletal [8] and cardiovascular systems [9]–[12], the brain [13], [14], and adipose tissue [15], [16], as well as their importance in male fertility [17]–[20]. Additionally, estrogen has been shown to work through both genomic pathways (transcriptional regulation through estrogen response elements) and non-genomic pathways (activation of cell-signaling cascades). Studies of a mouse model with ERα66 expressed only in the membrane [21] revealed the importance of genomic ERα66 in fertility.

In the past decade, it has become evident that there are at least two physiologically relevant splice variants of ERα66. The first, named ERα46 reflecting its molecular weight, was identified by Flouriot et al. [22] in the human breast cancer cell line, MCF7. The second, ERα36, was identified in 2005 [23]. Both ERα46 and ERα36 are truncated in the amino-terminus (173 amino acids) and lack the first transcriptional activation domain (AF1). ERα46 is identical to ERα66 in the remaining protein sequence. ERα36 lacks the second transcriptional activation domain (AF2) and has a unique C-terminus. The ERα36 protein is identical to the full-length receptor in its DNA binding domain and part of its dimerization and ligand binding domains. Because the ligand binding domain differs from that of ERα66, ERα36 may be capable of interacting with a wider array of estrogens than ERα66. Both ERα46 and ERα36 can form homodimers or they can heterodimerize with ERα66. ERα46 has a 2-fold higher binding affinity to the estrogen response element than ERα66 [24]. Post-translational modification by palmitoylation of ERα46 and myristoylation of ERα36 may target them to the membrane [23]. (For structure of the splice variants, see [25]).

These ERα splice variants have been studied primarily in human cancer cell lines. Their patterns of expression in mammalian organs and their contribution to estrogen signaling in the normal state are not known. It is evident that interactions among these receptors modulate estrogen signaling in the cell. An understanding of the tissue-specific expression of the various estrogen receptors is necessary for identifying the individual functional roles that each may play under physiologic conditions. As the kidney has been described as the most estrogen-responsive organ after gonadal tissue [26], it seems likely that ERα splice variants might contribute to the overall estrogen signaling in the kidney. Therefore, we hypothesized that the ERα splice variants and ERβ may be expressed differently in each organ in the body, and that estrogen receptors would be differentially localized to specific cell types within the kidney.

## Methods

### Ethics Statement

All animal procedures were approved and conducted in accordance with the guidelines of the Institutional Animal Care and Use Committee of the University of Nebraska Medical Center under protocols 10-081-12-FC and 10-082-10-FC.

### Animal Procedures

Mice were housed five per cage and in a 12-hr light/dark cycle environment. Mice had *ad libitum* access to water and standard mouse chow. Male and female mice were sacrificed under intraperitoneal ketamine (100 mg/kg)/xylazine (10 mg/kg) anesthesia at 6–12 wks of age and organs were harvested for western blotting and immunofluorescence. In the female mice, kidneys, heart, liver, uterus, mammary and ovaries were collected. In the males, the kidneys, heart, liver and testes were harvested.

### Western Blot (WB)

Tissue was snap-frozen in liquid nitrogen and stored at –80°C until homogenization and lysis in radioimmunoprecipitation assay (RIPA) buffer with Halt™ Protease and Phosphatase Inhibitor Cocktail (Thermo Fisher Scientific, Waltham, MA). Protein concentration was determined using the Bradford assay (Coomassie plus Protein, Thermo Fisher Scientific) with absorbance read at 595 nm on a uQuant microplate reader (Bio-Tek Instruments, Winooski, VT). After protein was boiled in 6X sample buffer (Boston Bioproducts, Ashland, MA), 33 µg were loaded on a 10% Tris-HCl gel (Bio-Rad, Hercules, CA). The gel was transferred to a PVDF membrane (Bio-Rad) and blocked in ODYSSEY blocking buffer (LI-COR Biosciences, Lincoln, NE) for 1 hr at room temperature. Then, membranes were incubated with primary antibodies in blocking buffer overnight at 4°C. The following morning, the membranes were washed in PBS with Tween 20, and then incubated in secondary antibodies for 1 hr at room temperature. After additional washes, membranes were visualized with the ODYSSEY® Infrared Imaging System (LI-COR Biosciences).

### Immunofluorescence (IF)

Kidneys were rinsed in saline, decapsulated and bisected longitudinally. They were then submerged in a Tissue-Tek® Cryomold tissue cassette filled with O.C.T.™ Compound (Sakura Finetek, Torrance, CA), frozen in liquid nitrogen and stored at –80°C. Sections (6-µm) were cut on a Leica CM3050S Cryostat (Leica Microsystems, Buffalo Grove, IL), transferred onto ProbeOn Plus® slides (Thermo Fisher) and fixed in 4% paraformaldehyde at 4°C. The sections were blocked for 1 hr in a buffer containing 10% donkey serum with BSA and Triton X-100. Then, the sections were incubated in primary antibodies in blocking buffer overnight at 4°C. The following day, after washing in PBS, the sections were incubated with fluorescent secondary antibodies in the dark at room temperature. Slides were mounted in ProLong® Gold Antifade Reagent with DAPI (Molecular Probes|Life Technologies, Grand Island, NY), coverslipped and visualized on a confocal microscope (Leica TCS SP5, Leica Microsystems) at 630× magnification.

### Antibodies

Rabbit anti-ERα36 (1∶150 IF females, 1∶50 IF males) was a kind gift of Zhao Yi Wang. Mouse anti-α smooth muscle actin (A5228; 1∶200 IF) was purchased from Sigma Aldrich (St. Louis, MO). Rabbit anti-ERα (H184, SC-7207; 1∶50 IF), rabbit anti-ERα (MC-20, SC-542; 1∶1000 WB), rabbit anti-ERβ (H-150, SC-8974; 1∶500 WB, 1∶50 IF), goat anti-synaptopodin (N-14, SC-21536; 1∶50 IF), goat anti-aquaporin 2 (C-17, SC-9882; 1∶50 IF) and goat anti-PECAM-1 (M-20, SC-1506; 1∶50 IF) antibodies were purchased from Santa Cruz Biotechnology (Santa Cruz, CA). The following antibodies labeled with Alexa Fluor® (AF) dyes were purchased from Invitrogen?Life Technologies: AF-488 donkey anti-rabbit IgG (A21206; 1∶200), AF-594 donkey anti-goat IgG (A11058; 1∶200), AF-594 donkey anti-mouse IgG (A21203; 1∶200) and AF-680 goat anti-mouse IgG (A21058; 1∶10,000 WB). Goat anti-rabbit IgG conjugated to IRDye® 800CW (611-131-122; 1∶10,000 WB) was from Rockland Immunochemicals (Gilbertsville, PA).

### Separation of Membrane and Cytosolic Fractions

The kidney was dissected into cortex and medulla. The cortex was then separated into membrane and cytosolic fractions using the Mem-Per kit (Pierce, Rockford, IL) according to the manufacturer’s protocol. Briefly renal cortices were homogenized in TBS, centrifuged, re-suspended in provided reagents, centrifuged again and then the membrane and cytosolic fractions were separated. The fractions were dialyzed overnight prior to proceeding with a standard Western blotting protocol.

### Statistics

Differences among groups were computed using two-way analysis of variance followed by Holm-Sidak method for pairwise multiple comparison analysis (Sigma Plot 11.0; Systat Software, San Jose, CA). One-way analysis of variance was performed within sex to account for unpaired reproductive tissue, followed by Tukey test. Probability values less than 0.05 were considered to be significant. Values are reported as means ± standard error.

## Results

### ERα66 in Male and Female Mice

**Figure 1:**
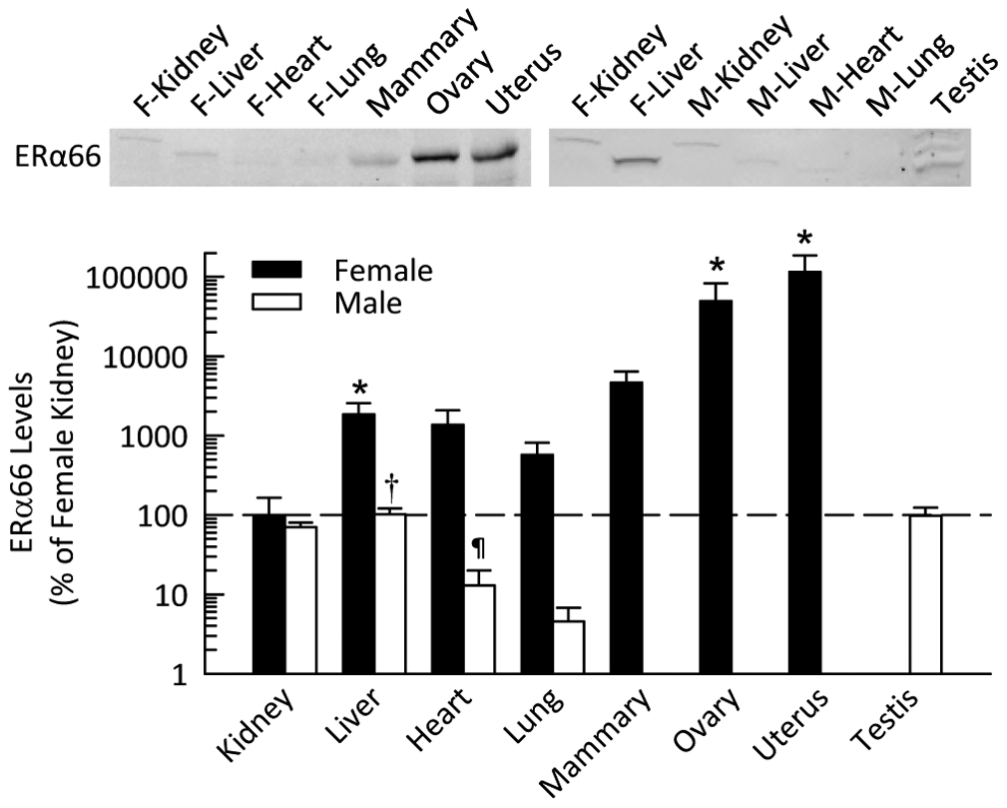
ERα66 protein level in various organs of mice. Shown are representative Western blots and quantification for expression in non-reproductive and reproductive organs harvested from female and male mice. Data are shown as percent of ERα66 in female kidney, plotted on a logarithmic scale. *P<0.05 vs. female kidney, **^†^** *P*<0.05 vs. female liver, ^¶^ *P*<0.05 vs. female heart (*n* = 4–5 per group).

Western blotting revealed that ERα66 protein level was more that 500-fold higher in the uterus and ovaries than in the kidney and other tissues; hence, the results are plotted in Figure 1 on a logarithmic scale. In the female mice, ERα66 expression was evident (in decreasing rank order) in the mammary gland, liver, heart, lung and kidney. In male mice, ERα66 protein level was highest in the liver, testis and kidney; however, the prominent band for testis on the Western blot ran at a slightly smaller molecular weight than evident in the uterus or ovaries. ERα66 protein expression was extremely low in the heart and lung of male mice.

### ERα46 in Male and Female Mice

**Figure 2:**
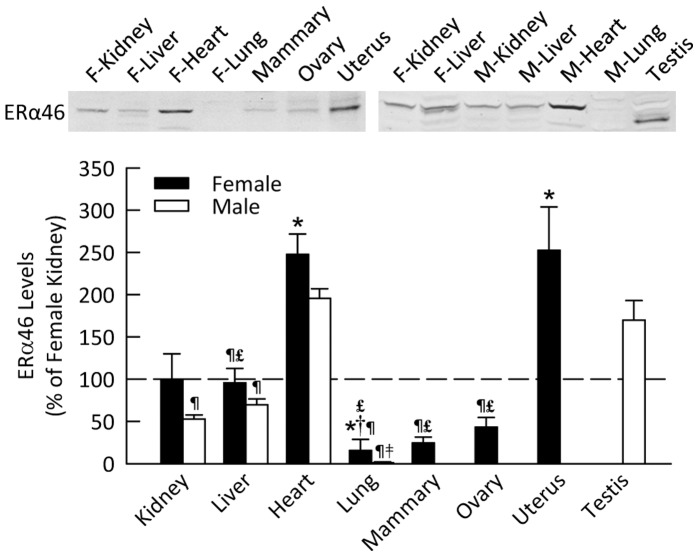
ERα46 protein level in various organs of mice. Shown are representative Western blots and quantification for expression in non-reproductive and reproductive organs harvested from female and male mice. Data are shown as percent of ERα46 in female kidney. *P<0.05 vs. female kidney, **^†^** *P*<0.05 vs. female liver, ^¶^ *P*<0.05 vs. heart (of same sex), ^£^ *P*<0.05 vs. uterus, ^‡^ *P*<0.05 vs. testis (*n* = 4–5 per group).

ERα46 protein level was most abundant in the heart and uterus in female mice (Figure 2), and in the heart and testes of male mice. As was the case for ERα66, the prominent band for ERα46 in the testis ran at a smaller molecular weight than in other tissues. ERα46 was present at lower levels in the remaining tissues investigated, although it was barely detectable in the lungs of both sexes.

### ERα36 in Male and Female Mice

**Figure 3:**
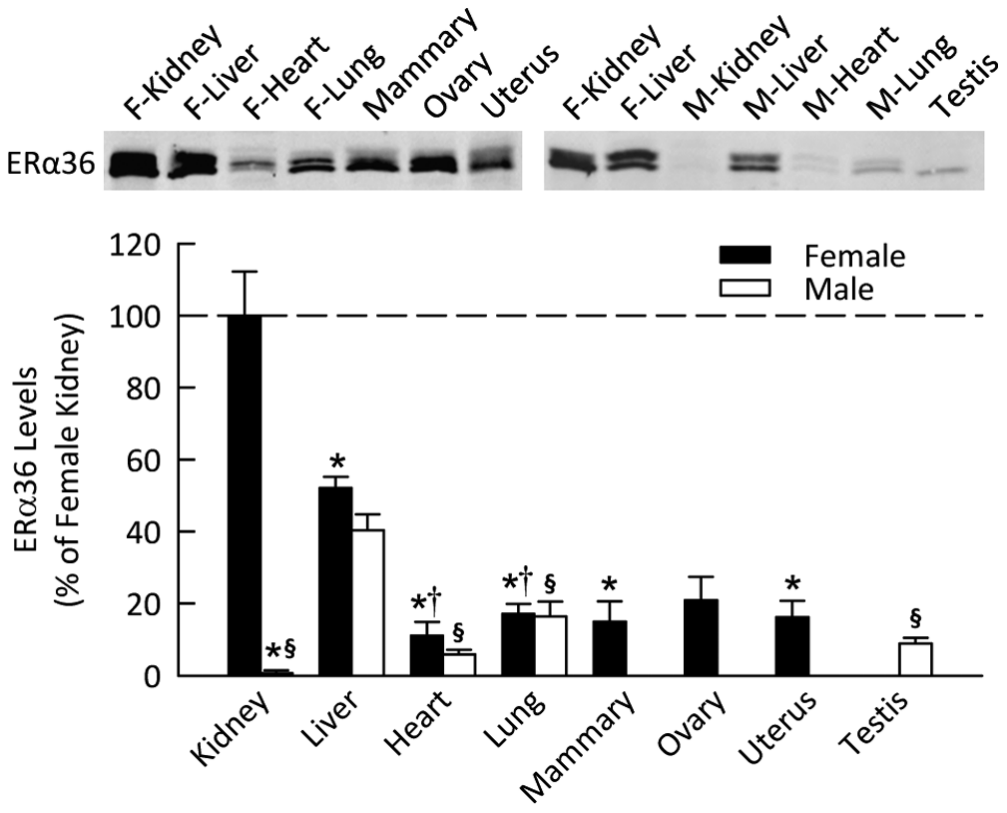
ERα36 protein level in various organs of mice. Shown are representative Western blots and quantification for expression in non-reproductive and reproductive organs harvested from female and male mice. Data are shown as percent of ERα36 in female kidney. *P<0.05 vs. female kidney, ^§^ *P*<0.05 vs. male liver, **^†^** *P*<0.05 vs. female liver (*n* = 4–5 per group).

As illustrated in Figure 3, ERα36 was most highly expressed in the kidney of female mice, but was almost non-detectable in the kidney of male mice (*P*<0.001 vs. females). In contrast, there was no sexual dimorphism with regard to ERα36 levels in other organs. This splice variant was expressed in all the tissues investigated but at significantly lower levels than in the female kidney, although the data for the ovary did not achieve statistical significance.

### ERβ in Male and Female Mice

**Figure 4:**
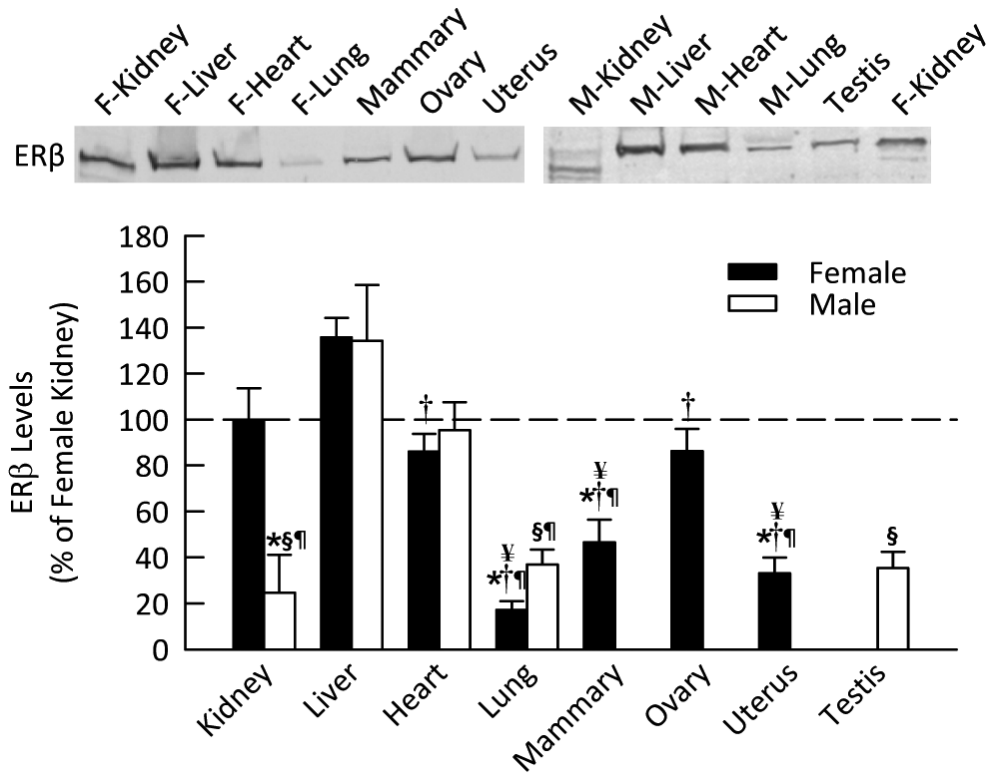
ERβ protein level in various organs of mice. Shown are representative Western blots and quantification for expression in non-reproductive and reproductive organs harvested from female and male mice. Data are shown as percent of ERβ in female kidney. *P<0.05 vs. female kidney, ^§^ *P*<0.05 vs. male liver, **^†^** *P*<0.05 vs. female liver, ^¶^ *P*<0.05 vs. heart (of same sex), ^¥^ *P*<0.05 vs. ovary (*n* = 4 per group).

In the female mice, ERβ protein level was highest in the liver, kidney, heart and ovary (Figure 4), with lower levels evident in mammary gland, uterus and lung. The male mice had similar expression of ERβ to that of females in all tissues except for the kidney, where levels in males were significantly lower than females. In the male mice, the liver and the heart had the most abundant level of ERβ.

### ER Localization in the Kidney

**Figure 5:**
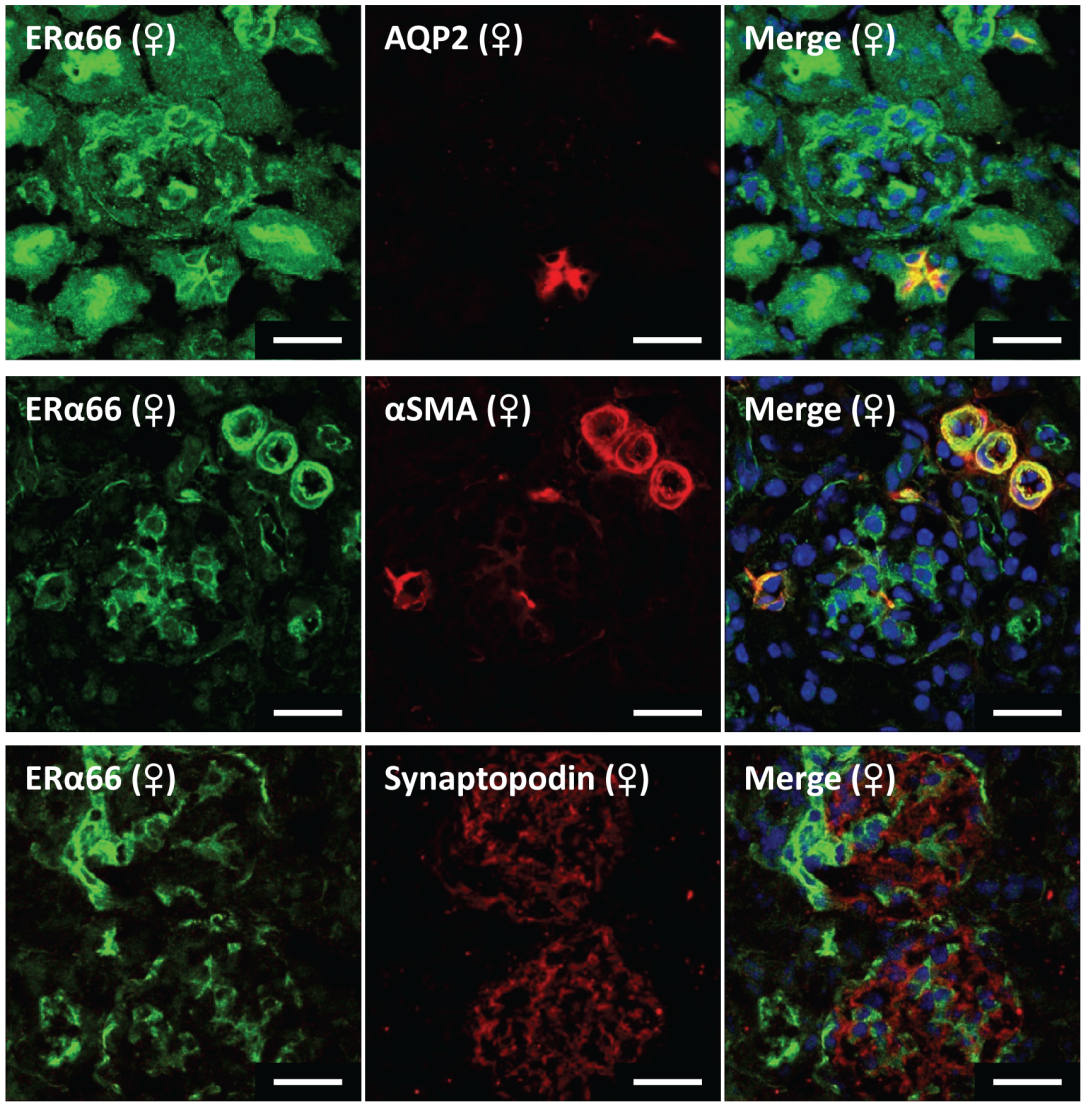
Representative immunofluorescence images localizing ERα66 in mouse kidney. Green fluorescence represents ERα66 in all images. Red fluorescence represents cell specific markers for co-localization (collecting duct marker, aquaporin 2; vascular smooth muscle & mesangial cell marker, α-smooth muscle actin; podocyte cell marker, synaptopodin). Nuclei are stained blue with DAPI. Scale bar = 20 µm.

Because the kidney has been described as the most estrogenic non-reproductive organ, intrarenal ERα66, ERα36 and ERβ localization was investigated in frozen sections. ERα46 localization could not be investigated because of the lack of a specific antibody for this splice variant. ERα66 detected using an antibody against the N-terminus was found mainly in the vasculature and in the glomerulus, although not in podocytes (Figure 5). In females, it was also detectable in the brush border of the proximal tubules and in the apical aspect of cortical collecting duct principal cells where it co-localized with aquaporin-2 (AQP2).

**Figure 6:**
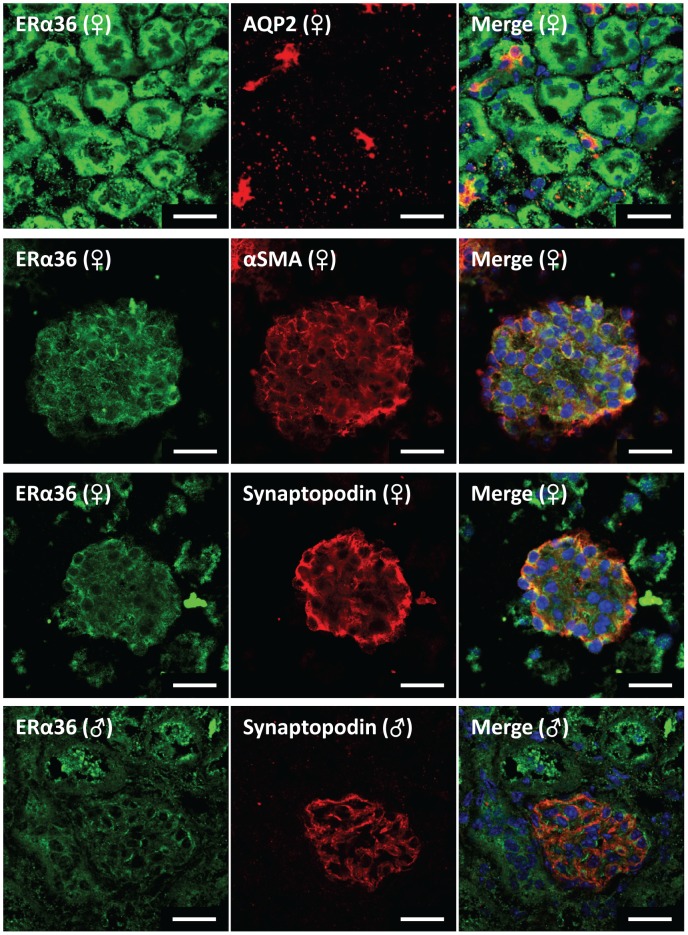
Representative immunofluorescence images localizing ERα36 in mouse kidney. Green fluorescence represents ERα36 in all images. Red fluorescence represents cell specific markers for co-localization (collecting duct marker, aquaporin 2; vascular smooth muscle & mesangial cell marker, α-smooth muscle actin; podocyte cell marker, synaptopodin). Nuclei are stained blue with DAPI. Scale bar = 20 µm.

As shown in Figure 6, ERα36 co-localized with the glomerular mesangial cell marker α-smooth muscle actin (αSMA) and was widely distributed in tubular epithelial cells, including the proximal tubule in both males and females. In females, ERα36 also co-localized with the podocyte-specific marker synaptopodin and the collecting duct principal cell marker AQP2; however, the data were inconclusive concerning its expression in vascular smooth muscle (not shown). ERα36 localization in males was similar to that evident in females, except for its failure to co-localize with synaptopodin in the glomerulus or α-SMA in vascular smooth muscle.

**Figure 7:**
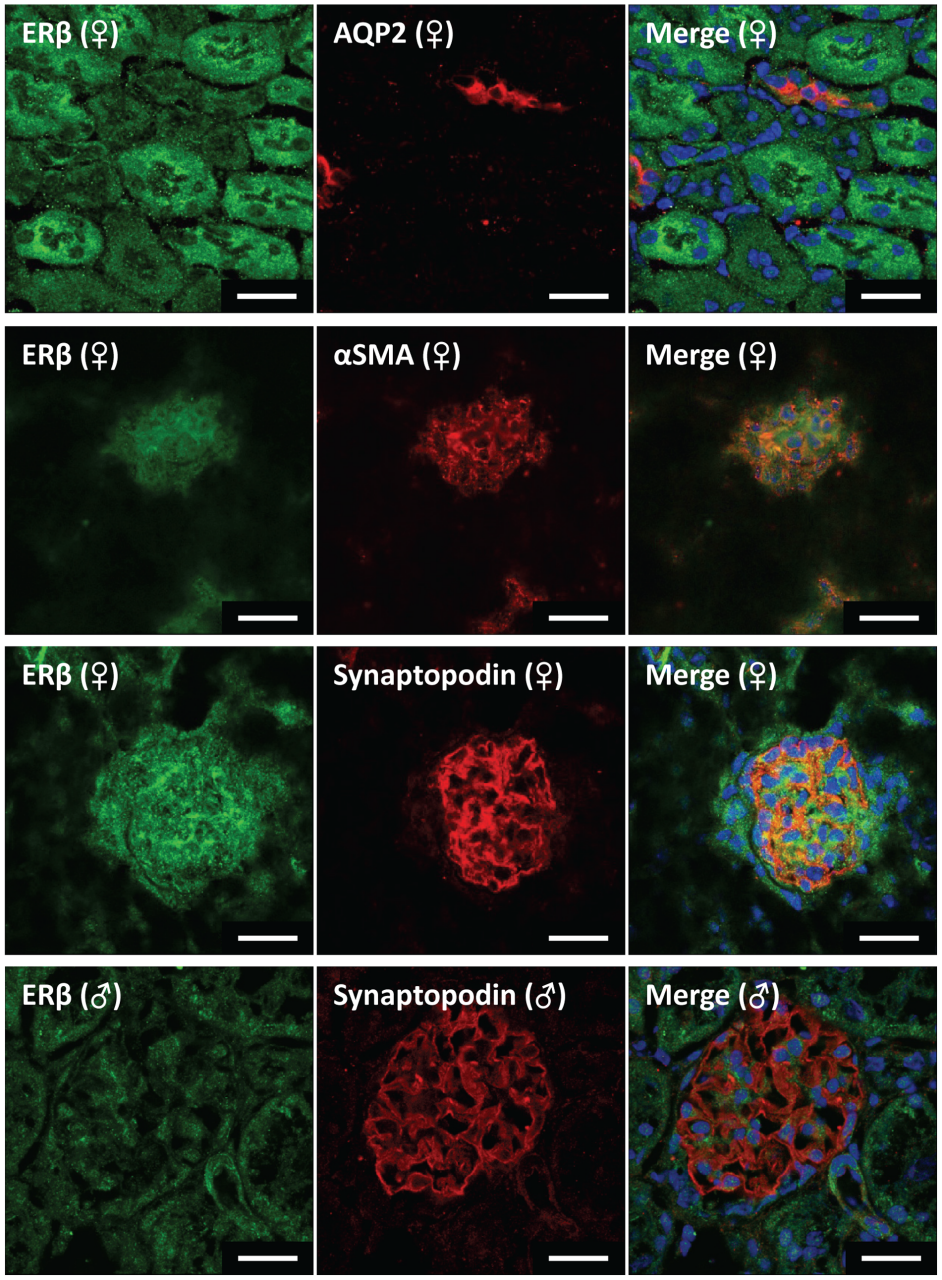
Representative immunofluorescence images localizing ERβ in mouse kidney. Green fluorescence represents ERβ in all images. Red fluorescence represents cell specific markers for co-localization (collecting duct marker, aquaporin 2; vascular smooth muscle & mesangial cell marker, α-smooth muscle actin; podocyte cell marker, synaptopodin). Nuclei are stained blue with DAPI. Scale bar = 20 µm.

ERβ co-localized primarily with the podocyte-specific marker synaptopodin in females but not in males (Figure 7). ERβ was also present in the mesangial cells as evidenced by co-localization with αSMA, although the mesangial localization appeared to be more nuclear in the male kidneys. ERβ immunostaining was more prominent in proximal tubular cells than in distal nephron segments including the cortical collecting duct, and was present in vascular smooth muscle in both males and females (data not shown).

**Figure 8:**
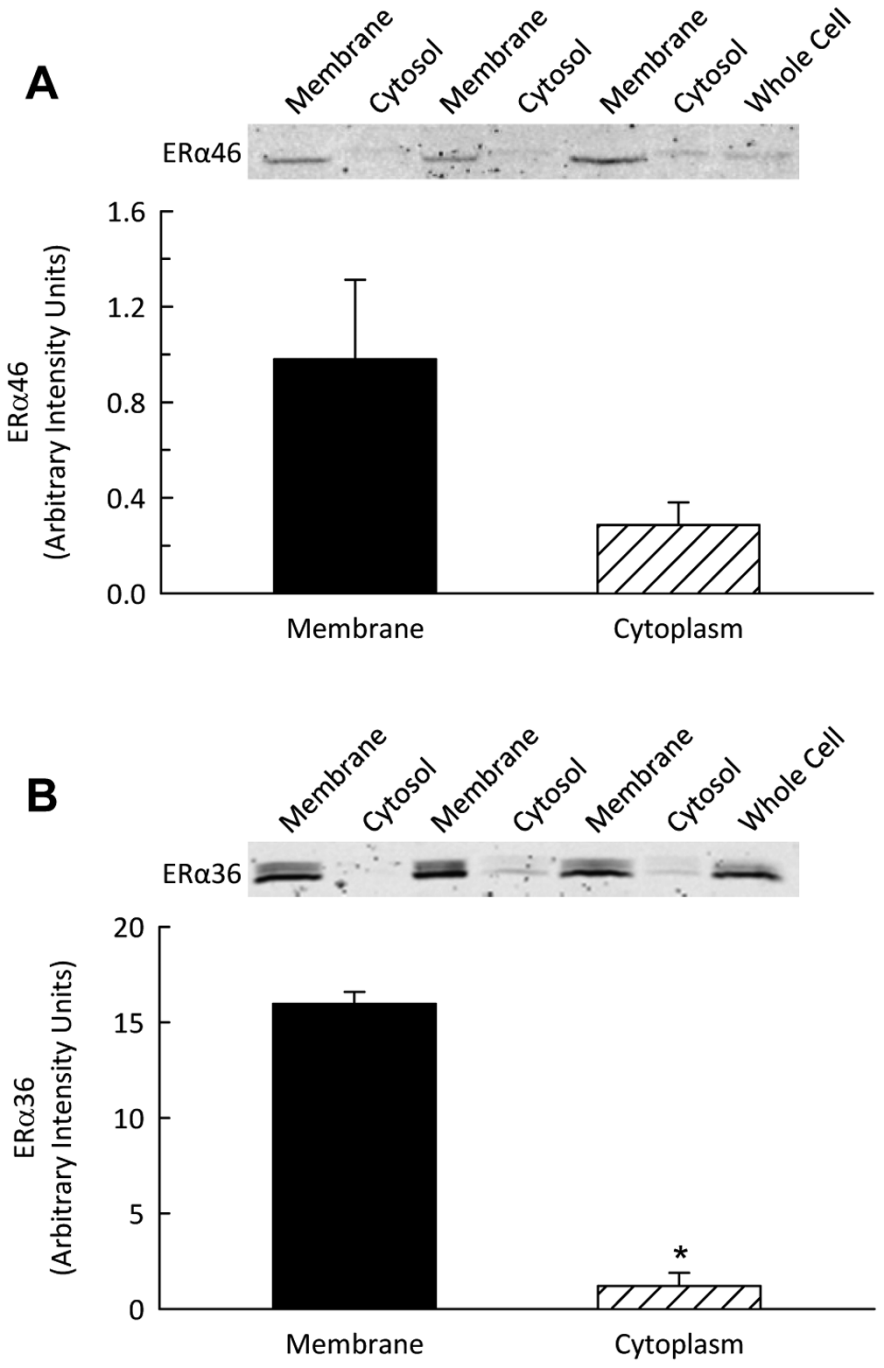
ERα46 and ERα36 protein level in membrane and cytosolic fractions from the renal cortices of female mice. Shown are representative Western blots and quantification for ERα splice variant expression in membrane and cytosolic fractions from the renal cortices from female mice. *P<0.05 vs. membrane fraction.

At the subcellular level, most of the renal ERα36 protein partitioned in the membrane fraction, with less than 10% in the cytoplasmic fraction (Figure 8). ERα46 was present in both the membrane and cytoplasmic fractions.

## Discussion

Expression of the various estrogen receptors differed widely among the organs of mice and also between males and females. The protein levels of ERα66 were low in all non-reproductive tissues. The highest expression was in the uterus and the ovaries. ERα66 expression was lower in males than in females in all the organs investigated, with levels in males averaging 70% of female levels in kidney, 6% of female values in liver, and 1% of female values in heart and lung. The bands on the Western blot were at a slightly higher molecular weight in the male and female kidney than the bands in other organs. The prominent band in the testis ran at a slightly lower molecular weight than that in the uterus and ovaries. This phenomenon has been reported previously by Baines [27], who suggested that this lower band was a degradation product. It is also possible that this represents a different isoform or that a post-translational modification present in the other tissues does not occur in the testis.

These data differ somewhat from previously reported results from Kuiper et al. [28], who detected high levels of ERα66 mRNA in the rat testis, male kidney and male adrenal gland, as well as the ovary and uterus. One explanation for the discrepancy is that their probes targeted the ERα DNA binding domain and, thus, would detect not only ERα66 but also ERα46 and ERα36. Additionally, the high levels of mRNA may not be translated into protein or may be rapidly degraded after translation and therefore not detectable by Western blot. It should also be noted that lower protein levels of ERα66 in the kidney do not necessarily equate with lack of activity. Indeed, despite its low relatively levels of expression in kidney, we and others have shown that loss of ERα66 results in physiologic changes in the kidney of female rodents [29]. This phenomenon likely reflects the ability of post-translational modification of proteins to alter function. In addition, post-translational modifications may interfere with antibody interaction at the epitope, thereby potentially impacting western blotting and immunofluorescence data.

In the present study using an antibody with an epitope in the N-terminus of the ERα66 protein, ERα66 was localized mainly to the vasculature and to the glomerulus. ERα66 was also found in the brush border of the proximal tubule and the apical aspect of collecting duct cells in female kidneys. The latter observations suggest a potential for ERα66 to modulate apical transport mechanisms in multiple segments of the nephron.

ERβ was present in all the tissues investigated. Males expressed ERβ levels similar to females in all organs except the kidney (25% of female value). These data again differ from the findings of Kuiper et al. [28], who did not detect ERβ mRNA in the male liver, kidney or heart. Similarly, Couse et al. [30] reported that ERβ mRNA was undetectable in the kidney of both male and female mice that were older than those used in the current study. Consistent with our observations, Catanuto et al. [31] have reported ERβ expression in the kidney. In a recent study in female rats, ERβ was detected in both the cortex and medulla [32]. The present studies revealed that ERβ was similarly expressed in the mesangial cells and vasculature of males and females. In contrast, prominent expression of ERβ was apparent in the podocytes of females but not males. Podocyte damage and apoptosis can underlie nephropathy and this sex difference in the expression of ERβ may contribute to increased severity of renal disease in males.

In both males and females, ERα46 protein level in the heart was approximately twice that found in the kidney. ERα46 was also highly expressed in the uterus and testes, but low in the mammary glands and ovaries. Recent studies have implicated ERα46 in both genomic and non-genomic estrogen signaling. ERα46 can inhibit transcription through the AF1 transactivation domain of ERα66, which may allow a proliferative phenotype [22]. Murphy et al. [33] demonstrated higher protein levels of ERα46 in differentiated macrophages than monocytes. Additionally, ERα46 in the membrane of endothelial cells can activate NOS3 [34]. Collectively these findings suggest that ERα46 can modulate the estrogenic effects of ERα66 in tissues that express both receptors, and that this occurs via both genomic and non-genomic mechanisms. Our observations suggest that this could occur in the heart and the uterus, which express both receptors; however, the extent of modulation might vary considerably due to the differences in the quantity of each receptor present in the tissue. This may be particularly true in the males that have low levels of ERα66 in the heart and testes. Esqueda et al. [32] reported a 46 kDa estrogen receptor in the cortex and medulla of female Dahl salt-sensitive and salt-resistant rats. Study of ERα46 is particularly difficult due to its identical protein sequence with ERα66. New methodologies need to be developed in order to differentiate the functional impact of ERα46 from that of ERα66.

ERα36 expression was low in all reproductive tissues investigated (mammary glands, ovaries, uterus and testes). ERα66 suppresses expression of ERα36 independent of estrogen, possibly through binding to the estrogen response element half site on the ERα36 promoter [35]. Therefore, it is not surprising that ERα36 expression is lowest in tissues known be predominantly regulated through ERα66. Even with low expression, however, ERα36 has been shown to be present in the plasma membrane of ovarian follicles and potentially influences non-genomic signaling [36].

The female kidney had the highest expression of ERα36, with the male kidney having the lowest expression of all organs studied (<1% of female kidney). ERα36 can exert a dominant negative effect by inhibiting ERα66 and ERβ transactivation through the AF1 and AF2 domains in the nucleus, but can also signal from the membrane [37]. This dominant negative role may be important in suppressing proliferative signaling through ERα66 in non-reproductive tissue. However, estrogen and estrogen receptor antagonists, such as tamoxifen and fulvestrant, can activate extracellular signal-regulated kinase (ERK1/2) signaling through ERα36 at the membrane, leading to proliferation [37]. This may suggest that genomic, rather than membrane-initiated, proliferative signaling is more prevalent in males.

In this study, the kidney was examined in greatest detail. Our findings suggest that the estrogen responsiveness of the female kidney may be primarily due to ligand binding to ERα36. In agreement with prior studies in cancer cells [23], ERα36 was distributed primarily in the membrane fraction of the renal cortex suggesting involvement in non-genomic signaling. Within the glomerulus, ERα36 co-localized with mesangial cells in both sexes and with podocytes in females only. We also found ERα36 present in the epithelial cells of the tubule. Thus, estrogen actions on a variety of renal cell types may involve non-genomic signaling via ERα36. The results of the present study also demonstrate sexual dimorphism of ER levels in the kidney, most strikingly with regard to the ERα36 splice variant but also for ERα46. Future studies are needed to elucidate the potential contribution of these receptors to sex differences in kidney function.

Pre-menopausal women have lower blood pressure than men, and the kidney is largely responsible for chronic regulation of blood pressure [38], [39]; however, the mechanism underlying this sexual dimorphism in blood pressure control is not completely understood. One obvious possibility is involvement of the vasodilator effect of estrogen, which arises through differential signaling mechanisms in various vascular beds of female rats [40]. Moreover, oxidative stress is more pronounced in healthy young men than in healthy, premenopausal women [41] and increases in women after menopause [42]. Estrogen receptor subtypes have been studied in the reduction of cardiovascular oxidative stress [12] and estradiol has been shown to attenuate superoxide production in an experimental model of hypertension [43]. In addition, male sex is a risk factor for nephropathy [44]. Estrogen has been shown to regulate matrix metalloproteinases which prevent collagen deposition in the glomerulus [45]–[47] and may contribute to the decreased risk in females. Fewer estrogen receptors in the male mouse kidney, particularly with regard to ERα66 and ERβ in podocytes, may render males more susceptible to apoptosis and glomerular injury. Finally, it is widely appreciated that female kidneys are smaller than male kidneys in the normal state, and evidence suggests that ERα66 is involved in compensatory renal growth after uninephrectomy in female mice [48]. Consistent with this observation, prominent proximal tubular brush border localization of this receptor could contribute to this phenomenon.

Viewed *en masse*, the results of the present study establish that estrogen receptor expression varies widely in different tissues. This new information expanding our understanding of which cells express each receptor can aid our interpretation of overall estrogen responsiveness in multiple tissues. In particular, the finding that the female kidney has the highest level of expression of ERα36 among the organs studied, in concert with an apparent sexual dimorphism in its intrarenal localization, should fuel future investigation to define the roles of this ERα splice variant in normal renal physiology and pathophysiology.
